# Progress in Medicine

**Published:** 1899-01-21

**Authors:** 


### PROGRESS IN MEDICINE.

### RENAL MEDICINE.

(*Continued from page* 263.)

**Malarial Nephritis.**—Thayer[10] finds that malaria, and especially the æstivo-autumnal form, frequently brings on Bright's disease. In 758 cases albumen was present in 321, and casts in 121, or nearly the same as in scarlatina. In yellow fever nephritis is still more common, and is here found on the first or second day, unlike what is seen in other diseases. No case of hæmaturia after the use of quinine had been seen by him at Baltimore. This is of special interest now owing to the discussion as to the causation of blackwater fever by the excessive use of quinine. Koch's opinion in favour of this theory has hindered the administration of quinine in malaria, and the matter is of such importance that a Government Commission has been ordered to investigate the origin of blackwater fever.

Nephritis without albuminuria is said by Edwards[11] to exist both in acute and chronic forms. The acute cases are undoubtedly rare, but have been observed by various authors, and instances are given here supported by post-mortem examinations. Scanty urine, with casts, and but little urea may be passed, and œdema or uræmic symptons may appear as well. Of course, albumen is often absent for a time in granular kidney, even if we do not recognise the pre-albuminuric stage which Mahomed postulated; but Stewart and others have also described special non-albuminuric types of Bright's disease, which Edwards confirms. The difficulty of diagnosis in these cases is undoubtedly great, and every symptom should be well weighed, but the writer thinks that they are more common than is supposed.

**Moveable Kidney.**—Max Einhorn[12] claims to have observed some 500 cases of this condition, which was well described by Rayer as long ago as 1836. He includes under the name any kidney which is more or less accessible to palpation. It clearly occurs more often in women than in men, and especially on the right side of the body. Indeed, during a given year he found it in 1·8 per cent. of his male patients and in 20 6 per cent. of the female ones, most of them being in attendance for diseases of the stomach. It nearly always occurs in connection with a displacement of other organs, and, therefore, although tight-lacing, parturition, &c., may assist in its production, Einhorn believes in an individual predisposition or type of structure as a necessary factor. Symptoms may be numerous and varied, or entirely absent. Among the more important ones affections of the stomach will require treatment specially directed to that organ, but for the pains caused by the actual movements of the kidney Einhorn acknowledges the importance of a well-fitting bandage. Indeed, by the use of this means, combined with a liberal diet, so that the patient puts on flesh, and by attention to the general health, and the cure of stomach complications, almost all cases can be satisfactorily treated. He is in general opposed to operative treatment, but in rare instances where all other measures fail, nephro-raphy may be resorted to. Another elaborate paper on the same subject appears by A. F. Galloway,[13] and lays stress on the effect of pregnancies, emaciation, spinal caries, and severe injuries in producing moveable kidneys.

**Cystitis.**—Max Melchier[14] believes that almost all cases of cystitis are due to microbial infection. In fact we usually find in any given case a nearly pure culture of some single species; most frequently this is the bacillus coli. With exception of one microbe, the proteus of Hanser, no organism can cause cystitis unless the bladder be affected by some injury or from the results of retention. On the other hand, these causes do not produce cystitis without the presence of microbes. In the majority of cases the urine is acid, and the cause is either the bacillus coli, the tubercle bacillus, a streptococcus, or the gonococcus. He finds nitrate of silver superior to everything else as a local remedy.

[10] Med. Rec., May 14. [11] Amer. Jour. Med. Sci.. Oct. [12] Med. Rec., Aug. 13. [13] Glasgow Med. Jour., Nov. [14] Med. Rec., July 30.

### CIRCULATORY SYSTEM.

**vular Disease.**—Arkle[1] states that of 1,000 cases of heart disease, 500 are affections of the mitral valve, 350 of the aortic, and the remainder of the right side of the heart. Rheumatic conditions, scarlet fever, measles, and chicken-pox are the commonest causes of endocarditis. The mitral valve is most exposed to strain, having to bear the whole force of the ventricular systole, and consequently suffers most frequently. Acute processes leading to softening of the tissues result in dilatation, chronic thickening processes to mitral stenosis. Between the simplest and the most malignant forms of ulcerative endocarditis there are all gradations. Advice is not generally sought in mitral regurgitant disease until compensation is just failing, cyanosis, shortness of breath, clubbing and glossing at the finger ends, and irregular action of the heart being the commonest symptoms. The pulse is irregular in force and rhythm. The systolic murmur may be mistaken for hæmic bruits, exocardial rub, or for the whiffing murmur caused by the expulsion of air from the little piece of lung overlapping the heart. Two diagnostic points are the accentuation of the second sound and the enlargement of the heart in its transverse diameter. Mitral stenosis may be of the "button-hole" or "funnel-shaped" variety. If unaccompanied by regurgitation the left ventricle is small in marked contrast with the right. Embolism from clots formed in the dilated auricular appendix is common in stenosis. There is frequently præcordial bulging, and the first sound may become so modified as to be mistaken for the second. The murmur should be timed by placing the finger upon the impulse. At a later stage the second sound may be inaudible at the apex, and the presystolic and diastolic murmurs may disappear. From the progressive nature of the disease prognosis is unfavourable. A review of the spasmodic affections of the mitral orifice is given by Barie.[2] Stenosis or regurgitation may occur. Stenosis of spasmodic origin is most common amongst anæmic, hysterical girls, and the symptoms and physical signs disappear with an improved state of the blood. Such cases have been described by Laënnec, Peter, Picôt, Andéoude, and

Jacot - Descombes. Since Stokes first described spasmodic mitral incompetence many cases of regurgitation of this nature have been recorded. The symptoms of spasmodic mitral affections are generally accompanied by the stigmata of hysteria and by well-marked spasmodic vascular disturbances. They vary from day to day, and are easily intensified by mental or physical exertion. They are seldom permanent, and the prognosis is favourable. Mitral lesions of spasmodic origin present the same physical signs as organic lesions. The possibility of spasmodic contraction of the heart muscle wall has been demonstrated by many observers, and it is certain that bruits can be caused by this means. Some have explained the phenomena as being due to ill-regulated contractions of the musculi papillares, or of the muscular fibres inserted into the fibrous ring of the left auriculo-ventricular orifice. At the commencement of an acute attack of endocarditis, spasmodic affections of the cardiac orifices are frequently observed. The subject is deserving of further study. It is associated closely with secondary spasm (breathlessness, palpitation, præcordial pain), such as is met with in cases of organic heart disease. Cases of pulmonary regurgitation are extremely rare, and the patient shown by Maclure at the North-West London Medical Society[3] attracted much attention and comment. There was a to and fro murmur, loudest over the pulmonary area, and conducted down to the left of the sternum. Apex beat in fifth space in nipple line. There were also evidences of mitral disease. The absence of Corregan's pulse, capillary pulsation, and of much hypertrophy and dilatation of the left ventricle, pointed to pulmonary rather than aortic disease. A case illustrating the difficulty of differentiating between valvular disease and muscle failure of the heart is recorded by Steele.[4] There was dyspnœa, dropsy, engorged liver and spleen, with urine of low specific gravity containing much albumen. The area of cardiac dulness was much increased, systolic murmurs at tricuspid and mitral orifices, the later not conducted to the back. Pulse irregular. Occasionally the cardiac murmurs disappeared for a short time. Post mortem, both ventricles were dilated and hypertrophied, mitral valves indurated, orifice contracted, tricuspid orifice dilated. The stenosis of the orifice and the fact that the systolic murmur occasionally disappeared negatived mitral regurgitation. The irregular pulse and regurgitant murmurs pointed to muscle failure, and the large quantity of albumen in the urine, together with the discovery after death of early interstitial changes in the kidneys also pointed to this diagnosis. At the same time large quantities of albumen may be found in heart cases without kidney complication, whilst with advanced granular disease of the kidney only a trace may be present. The case was probably one of mitral stenosis complicated with chronic interstitial nephritis.

